# Can Green Walls Reduce Outdoor Ambient Particulate Matter, Noise Pollution and Temperature?

**DOI:** 10.3390/ijerph17145084

**Published:** 2020-07-14

**Authors:** Naomi Paull, Daniel Krix, Fraser Torpy, Peter Irga

**Affiliations:** 1 Faculty of Science, School of Life Sciences, University of Technology Sydney, Sydney 2007, Australia; Naomi.Paull@uts.edu.au (N.P.); Daniel.Krix@uts.edu.au (D.K.); Fraser.Torpy@uts.edu.au (F.T.); 2 Faculty of Engineering and Information Technology, School of Civil and Environmental Engineering, University of Technology Sydney, Sydney 2007, Australia

**Keywords:** green walls, air quality, noise pollution, urban heat island, traffic density

## Abstract

Green walls have previously demonstrated the capacity to reduce particulate matter (PM), noise pollution, and temperature conditions in manipulative experiments and computational models. There is, however, minimal evidence that green walls can influence ambient environmental conditions, especially taking into account the variable environmental conditions encountered in situ. The aim of this paper was to determine if green walls have a quantitative effect on ambient air quality in an urban environment. Ambient PM, noise, and temperature were recorded at 12 green wall and adjacent reference wall locations across a dense urban centre, over a 6-month period. The results indicated that PM levels and temperature did not significantly differ between the green wall and reference wall sites. Ambient noise at the green wall sites, however, was significantly lower than at the reference wall locations. It is suggested that mechanically assisted, or ‘active’ green wall systems may have a higher PM and temperature reduction capacity, and if so, they will be more valuable for installation in situ compared to standard passive systems, although this will require further research.

## 1. Introduction

The proportion of people living in dense urban areas increased from 34% in 1960 to 54% in 2014 [1], with living in cities increasingly correlating with a range of health problems [2]. Diminishing air quality in dense urban environments, in particular, is an emergent health problem [3,4,5]. It has been suggested that more than 1.78 billion people have inhaled polluted air over the last decade [6], with an estimated 7 million deaths from air pollution exposure in 2012 [7]. Air pollution is comprised of a combination of gases and solid and liquid particles, and is sourced particularly from vehicle exhaust, dust, and industrial emissions [7]. Smaller sized particles penetrate deeply into the lungs and alveolar regions, making them especially dangerous to human health [3,8,9,10]. Furthermore, as urban areas become increasingly dense, issues such as excess heat and noise are produced [11], which negatively impacts wildlife, vegetation, and human populations; altering local climate and increasing building energy demands [7,12]. As such, technologies that reduce exposure to, and mitigate the effects of the factors associated with dense urban environments—air pollution, the urban heat island effect, and noise pollution—are paramount.

The capacity of plants and their associated growing substrates to effectively clean the air, produce cooler ambient temperatures, and reduce ambient noise has been demonstrated [13]. The amount of space for green areas such as parks within cities, however, is rapidly declining [13]. It is thought that at least 80% of buildings within cities will still be in use by 2050 [14], making the implementation of green walls onto pre-existing building surfaces a space-efficient urban greening initiative. Vertical greenery utilises plants which are grown in small pots, planter boxes, or specially designed surfaces, and are hung vertically on walls [15]. Green walls are thought to be capable of positively impacting the urban environment in many ways including: mitigating air pollution [16,17,18], decreasing surface temperatures [19,20,21,22,23], and reducing noise [24].

Vegetation acts as a particulate sink, [25], due to plant surfaces acting as a source of turbulence and increasing turbulent diffusion, influencing PM diffusion and sedimentation [26,27]. Green walls have been proposed as an appropriate tool to reduce PM via deposition without altering the air exchange between the street canyon and the air above it [28]. Past research has shown a positive impact of vegetation on ambient air pollutant removal. Irga et al [29] recorded lower PM concentrations in areas of Sydney which had abundant tree vegetation; while Al-Dabbous and Kumar [30] noted that roadside vegetation had a significant, wind dependent effect on nanoparticle concentrations in the UK; and [31] detected significant effects of Spanish peri-urban forests on ambient air quality. PM reduction by green walls, and an overall improvement in local air quality has also been noted in previous studies [8,10,16,32,33]. However there remains some uncertainty regarding the capacity of plants to effectively remove ambient PM pollution. Wind strength, the presence of buffer zones, the distance from the pollution source, and particle quality all affect the distribution of pollutants [34]. Leaf area index, humidity and street canyon geometry are also influential on vegetation pollutant removal [35,36], making it difficult to draw general conclusions, as these factors may vary both temporally and spatially.

The number of people exposed to noise pollution in urban areas continues to increase due to the expansion of transport, residential areas, and infrastructure [37]. Noise pollution is common and more frequent in dense urban environments due to the close proximity to an array of continuous noise emitting sources [38], including transport (road, rail and air), industry, construction, public works, and neighbourhood related noise [37]. Of these sources, it has been suggested that >70% of unwanted sound in urban Australia is from road traffic [39]. Exposure to excessive noise can have negative impacts on human health and well-being [12]; as it disrupts sleep and work productivity, limits cognitive function, contributes to mental illness, and can even cause cardiovascular disease [40]. The hard surfaces of street canyons reflect sound, increasing overall urban ambient noise [41].

Unlike normal building surfaces such as steel, concrete, and glass [7], plant structures can absorb the noise that would otherwise be reflected between buildings [42]. This effect is due to mechanical vibrations of plant elements induced by sound waves, leading to dissipation by converting sound energy to heat [43,44,45]. Additionally, the thermo-viscous boundary layer at vegetation surfaces assists with sound reduction [24], and sound energy can be effectively reduced by the destructive interference of sound waves [46]. Sound can also be reflected and scattered (diffracted) by plant trunks, twigs, branches, and leaves [46]. The presence of soil or soil-like substrates can lead to destructive interference between the direct contribution from the source to the receiver and a ground-reflected contribution [24], an effect referred to as the ‘acoustical ground effect’ or ‘ground dip’ [24]. Plant roots and litter lead to an acoustically very soft (porous) soil [46], resulting in a distinct shift towards lower frequencies [47]. This ground dip is especially effective at limiting typical engine noise frequencies (approximately 0.1 kHz) [46]. Leaves, alternately, produce a sound absorption effect predominantly in the high frequency range (>1 kHz), whilst the wooden parts of vegetation (i.e., branches, twigs and stems) have a sound absorption effect in the mid frequency range (0.5–1 kHz) [48]. Thus, whilst the capacity of vegetation to absorb noise has been documented, it is of interest whether these effects can be detected in ambient noise pollution proximal to green walls, across various locations and several months.

The heavy use of glass facades, concrete sidewalks, steel surfaces, and asphalt roads lead to the radiation of heat rather than absorption [7]. Thus, urban areas tend to have much higher temperatures than surrounding rural and peri-urban areas, this phenomenon being termed the ‘urban heat island’ effect [7]. Increases in urban heat can result in increased air pollution levels, altered rain and wind conditions, increased energy demands, poor run off water quality, increased cooling costs, heat related illnesses, and mortality rates [7]. Urban vegetation can help reduce ambient air temperature through evapotranspiration and shading [49,50], due to leaves absorbing ambient heat energy through the process of photosynthesis and shading [51]. As urban heating effects are highly dependent on the geographical, morphological, and climatic conditions of an area [52], it is important to examine the effect of green walls on temperature reductions over a range of spatial and temporal environments to uncover their true potential.

Most green wall studies have been limited to Europe and Asia, resulting in insufficient testing across different green wall systems and varying climatic and pollutant conditions [15,53]. Although the pollution reduction potential of green walls has been documented [16,33,36], evidence for air pollution reduction by green walls in the built environment at a local scale is limited [27]. Additionally, most studies on green wall PM removal assess removal on a leaf scale, followed by modelling to generalise findings to an ambient scale [15], which have rarely been empirically validated. The current work investigated ambient PM, temperature, and noise conditions at 12 green wall and spatially matched reference wall locations across Sydney over a 6-month duration, and thus aimed to empirically quantify the effectiveness of existing green walls at ecosystem service provision.

## 2. Materials and Methods

### 2.1. Measurement Sites

**Figure 1:**
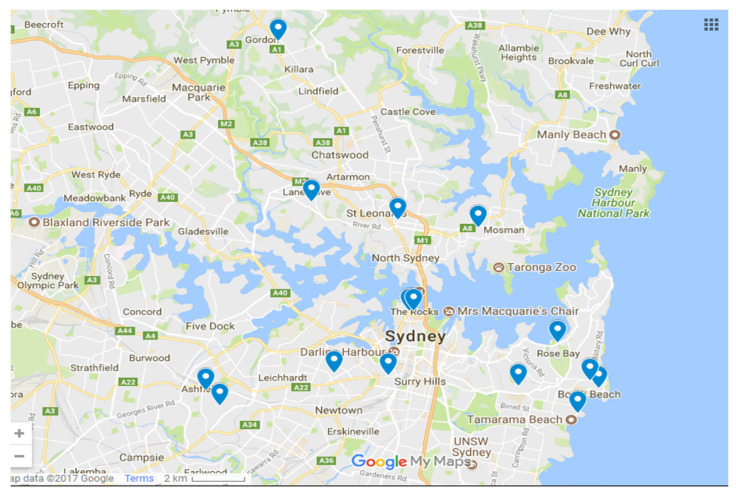
Spatial distribution of the study sites (image: Google maps).

Twelve sites within the urban Sydney region were selected based on the presence of structurally similar outdoor green walls. The sites varied in location, use, and pollutant conditions (Table 1; Figure 1; Appendix Table A1).

Sydney, Australia has a population of 5.2 million and lies on a coastal lowland plain between the Pacific Ocean and elevated sandstone tablelands. Sydney’s climate is warm and temperate, and rainfall is fairly evenly distributed throughout the year. Sydney city’s air quality is generally comparatively good, although PM exceeds the national standards on occasion, especially during bushfires. Noise pollution in Sydney is of concern, with Sydney having the highest traffic related noise exposure amongst Australian cities.

### 2.2. Measurement Method

Air quality, traffic density, noise and temperature assessments were conducted monthly for 6 consecutive months at all sites between June 2017 and November 2017. The order in which sites were sampled was randomised amongst months to eliminate systematic variation. Samples were taken at green walls and reference walls on the same visit, within 30 min of one another. Samples were not taken on rainy days, as rainfall removes PM from the air [54], and no bare soil was present within 30 m of the sampling locations so as to not artificially spike ambient PM concentrations. Average monthly weather variables were collected for each site using Australian Bureau of Meteorology data to account for weather dependent correlations with in situ conditions. Green wall area and plant number were also recorded for inclusion in the statistical analysis.

At each site, a reference wall was selected based on the following criteria: the reference wall was exposed to the same traffic pollution source as the green wall; the reference wall had similar building characteristics to the green wall, and the reference wall was within 10 m of the green wall. These criteria were used in an attempt to eliminate confounding influences effecting the variables between wall types.

PM measurements were conducted using a DustTrack II 8532 laser densitometer (TSI, Shoreview, MN, USA; sensor type: 90° light scattering, accuracy: ±5%). At each site, time weighted averages for two PM size fractions (particulates <10 µm in diameter—PM_10_; and particulates <2.5 µm in diameter—PM_2.5_) were collected between 10:00 a.m. and 3:00 p.m. at both green wall and reference wall locations at each site. At each site location and for each wall type, one 3 min time weighted average sample was collected (an average of each air reading taken per second for 3 min). This was done once per month for a 6-month duration. Samples were taken at 1.5 m above ground, and 0.5 m from the walls.

Traffic density was predicted to be the predominant PM source at all sites. Most sites were situated near residential properties, academic institutes or highways; away from industrial sources of pollutant emissions other than minor infrastructure work. Traffic density was quantified at the closest intersection to the sites for a 30 min duration, each month. Days of the week and times at which air quality and traffic density tests were conducted were limited to weekdays between 10:00 a.m. and 3:00 p.m. to avoid peaks caused by work and school commuters and randomized amongst sites and months [29].

Noise and temperature readings were taken at four point sources across both the green and reference walls at each site using a Digitech Multifunction Environment Meter (sensor type: thermo and audio sensor; accuracy: ±1.2%). The temperature of the ambient air was measured 0.5 m from both wall types. The average and standard error were then determined from the point samples.

### 2.3. Statistical Analysis

Mean values were calculated for PM_2.5_, PM_10_, noise, temperature, and traffic for each month of the study at each site. For use in subsequent analysis, differences between the reference walls and the green walls (henceforth ∆ values) for PM_2.5_, PM_10_, noise and temperature were calculated as the (reference wall value—green wall value), so that higher values of ∆ indicate higher PM, noise or temperature levels at reference walls relative to the green walls. Prior to analysis, ∆ PM_2.5_ and ∆ PM_10_ were square root transformed to satisfy the assumptions of the models, with a negative sign given to untransformed values less than zero. After transformation, observations retained their original sign (i.e., positive values were not made negative, and vice versa), while decreasing the deviation of the ∆ PM values.

To test if a systematic difference in ambient PM concentrations existed between the green and reference walls, one-tailed paired-sample *t*-tests were used. Following this, the relationship between ∆ PM_2.5_ and ∆ PM_10_ was tested using a linear mixed model regression (LMM) with ∆ PM_10_ as the response, and ∆ PM_2.5_ as the predictor variable, with a random slope between ∆ PM_2.5_ and ∆ PM_10_ and a random intercept fitted for each site. Site level differences in ∆ PM were then examined, by fitting a linear model to the ∆ PM data and using site as predictor (categorical fixed factor, 13 levels). Using a joint test, the coefficients produced by this model were then tested for differences from zero. Finally, to understand the relationship between PM and environmental factors, multiple regression models of green wall PM_2.5_ and PM_10_, and ∆ PM_2.5_ and ∆ PM_10_ were built using traffic density, wind, humidity, green wall size, and the number of plants used in the green walls as predictors. Here, LMMs were again employed, with a random intercept fitted for each site.

Using a similar approach to that for PM, overall differences in noise and temperature between green and reference walls were first tested using one-tailed, paired *t*-tests. Site level differences from zero in ∆ noise and ∆ temperature were then tested by fitting a linear model to each response using site as predictor (categorical fixed factor, 13 levels). Where the joint test of these models was significant, the coefficients from the models were tested for differences from zero to determine which site or sites generated the difference.

All analyses and visualisation were performed in R 3.6.1 (R Core Team 2014, Vienna, Austria), using the packages lme4 [55], lmerTest [56], emmeans [57], and car [58]. Where LMMs were used and models did not include categorical terms, degrees-of-freedom were approximated using the Satterthwaite method for *t*-tests, while LMMs containing categorical terms used Wald Chi-square tests to generate ANOVA tables.

## 3. Results & Discussion

### 3.1. Differences in PM Concentration between Wall Types

**Figure 2:**
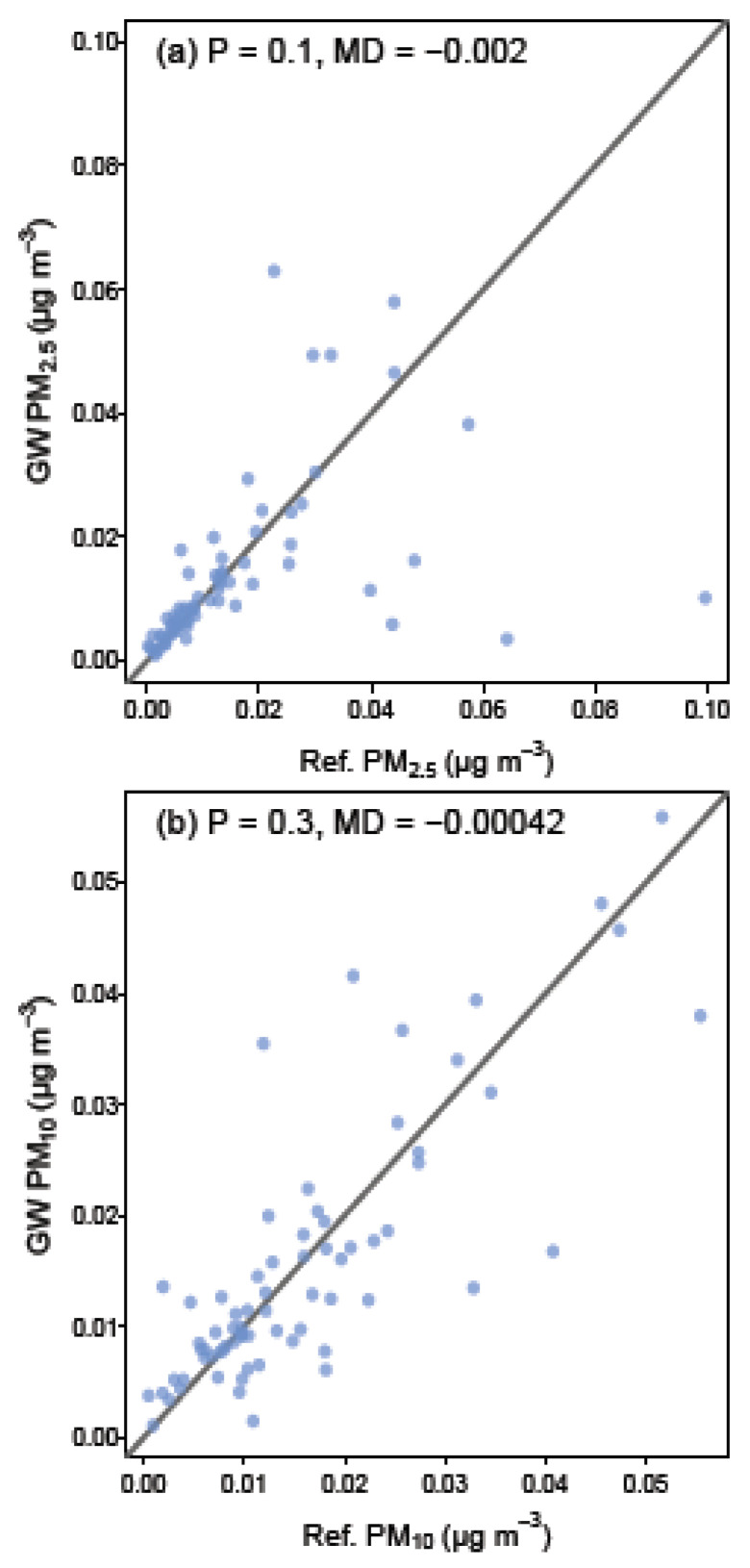
Scatter plots showing concentrations of PM_2.5_ (**a**), and PM_10_ (**b**) at the green wall (*y*-axis) and matched reference wall sites (*x*-axis). The black lines in (**a**,**b**) represent 1:1 relationships, therefore all points falling below the line indicate lower values at the green wall relative to the reference wall, and the converse for points above the line. *p*-values for the paired *t*-test and the mean difference (MD, green wall value—reference wall value) between paired samples are shown at the top of the graphs.

The ambient PM concentrations at green wall and reference wall locations are presented in Figure 2. Average PM concentrations at the green wall sites were not significantly lower than those recorded at the paired reference walls for PM_2.5_ (t_71_ = −1.10, *p* = 0.1; Figure 2a), nor PM_10_ (t_71_ = −0.50, *p* = 0.3; Figure 2b). Additionally, no significant association between ∆ PM_2.5_ and ∆ PM_10_ was found (t_10.4_ = 1.93, *p* = 0.08; Figure 2). The common method for determining PM reductions in previous research has been computational modelling, making comparisons with the current in situ results difficult. To the authors’ knowledge, there are no standard methodological guidelines surrounding in situ PM monitoring, and qualifications for what is required by ‘reference walls’. As such, it is unknown whether the 10 m distance between the green wall and reference wall locations was sufficient to observe PM reductions specific to the green walls. Furthermore, it was not appropriate to compare green and reference walls that were any further than 10 m apart as both traffic source and building type altered too significantly at these greater distances, which would have confounded PM comparisons. It is nevertheless suggested that future studies use greater distances between wall types if it is possible to maintain representative conditions over such distances. Whilst it is very likely that there would be a strong relationship between green wall PM efficiency and distance from the wall surface, the current work standardized sampling at 0.5 m from each wall type as distances further than this would have resulted in monitoring in the middle of busy roads at many sites. PM deposition however, has been previously found to be unrelated to differing heights [33] and as such PM was monitored at normal chest height.

This result was surprising, as previous green wall studies have predicted high pollutant removal capacities [27,35,59]. This removal capacity is thought to be due to leaves creating turbulence, forcing compaction between aerosolized PM particles, leading to deposition and eventual accumulation of PM on the leaf surfaces [27,60]. Whilst some PM accumulation was apparent in the current study from visual inspection of the leaves, it is clear that the level of PM removal exhibited had a general effect on proximal air quality that was too small to be detected using the current method, as has been the case in some previous work [61].

The inability of our methods to detect PM removal may have been related to the characteristics of our reference samples. Solid walls have pollution dispersion patterns that may be of similar magnitude to green walls [62,63], with structures such as noise barriers and low boundary walls being shown to have quantifiable effects on proximal air quality [64]. It is also possible that the proximity of the green and reference walls at all sites was such that the influence of the green walls on ambient air quality was sufficient to affect the matched reference sites. It is thus suggested that further studies aim to examine the spatial extent to which air pollution mitigation effects range from their sinks, especially when novel systems are being tested. Alternatively, before–after studies may be of value, as has been proposed previously [27]. Controlling for temporal effects with such methods, however, will be challenging. Due to this limitation, some previous studies have used wind-tunnel and/or modelling trials to replicate before-and-after comparisons e.g., [65]; albeit with limited ability to replicate key in situ influences such as weather and traffic patterns [27].

Currently, claims for significant pollution removal by green walls are matched by many that propose that the effect of urban vegetation on local air pollution mitigation has been exaggerated [66,67,68,69,70]. Thus, there clearly remains uncertainty regarding the pollutant removal capacity of green walls, and as such, further studies are required to identify the true role vegetation plays on local air pollution mitigation [71].

Whilst the current study detected no significant PM removal by green walls, previous controlled laboratory trials [72,73,74,75] have shown that PM, volatile organic compounds (VOCs) and CO_2_ can be removed at a considerably greater efficiency with the conversion of passive green walls to active systems. Active green wall systems utilise assisted aeration using some form of mechanical fan to actively force air through the plant root and substrate membrane [76]. This leads to an increased surface area for PM adherence, leading to PM entrapment within the substrate and plant root matrix, thus filtering the air more effectively than through the simple diffusion mechanisms on which passive systems are reliant [77,78,79]. Therefore, it is suggested that future studies focus on the potential effect active green wall systems have on ambient PM conditions in situ.

**Figure 3:**
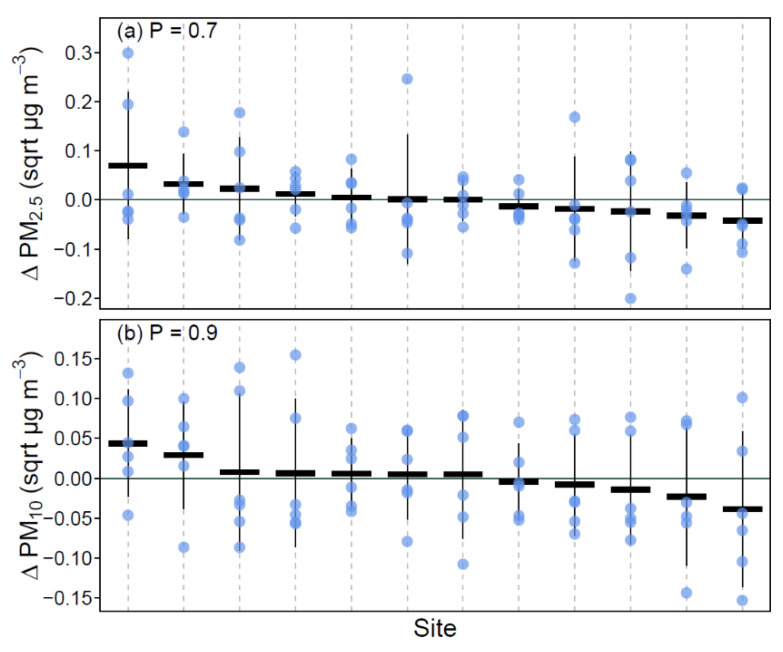
Plots of ∆ PM_2.5_ (**a**) and ∆ PM_10_ (**b**). Blue points show the ∆ PM values, thick black horizontal lines represent the means for the sites, and thin vertical black lines the 95% confidence interval of the mean for the sites. The *p*-value at the top of each plot is the result from a joint test of model coefficients with the null hypothesis that the sites do not differ from zero. Sites are sorted on the *x*-axis by their mean value (highest to lowest) for ease in interpretation. The solid horizontal line indicates zero on the *y*-axis, representing equal values of PM at the green wall and reference wall.

Site-specific PM patterns were investigated to determine if any site-specific PM sources were influential on the relationships observed between PM at the green walls and reference wall locations at each site. This analysis indicated that there was no significant difference from zero found in ∆ PM_2.5_ amongst sites (F_12,60_ = 0.72, *p* = 0.7; Figure 3a), with similar results for ∆ PM_10_ (F_12,60_ = 0.52, *p* = 0.9; Figure 3b). These findings are likely due to the relatively stable pollution conditions within central Sydney.

**Figure 4:**
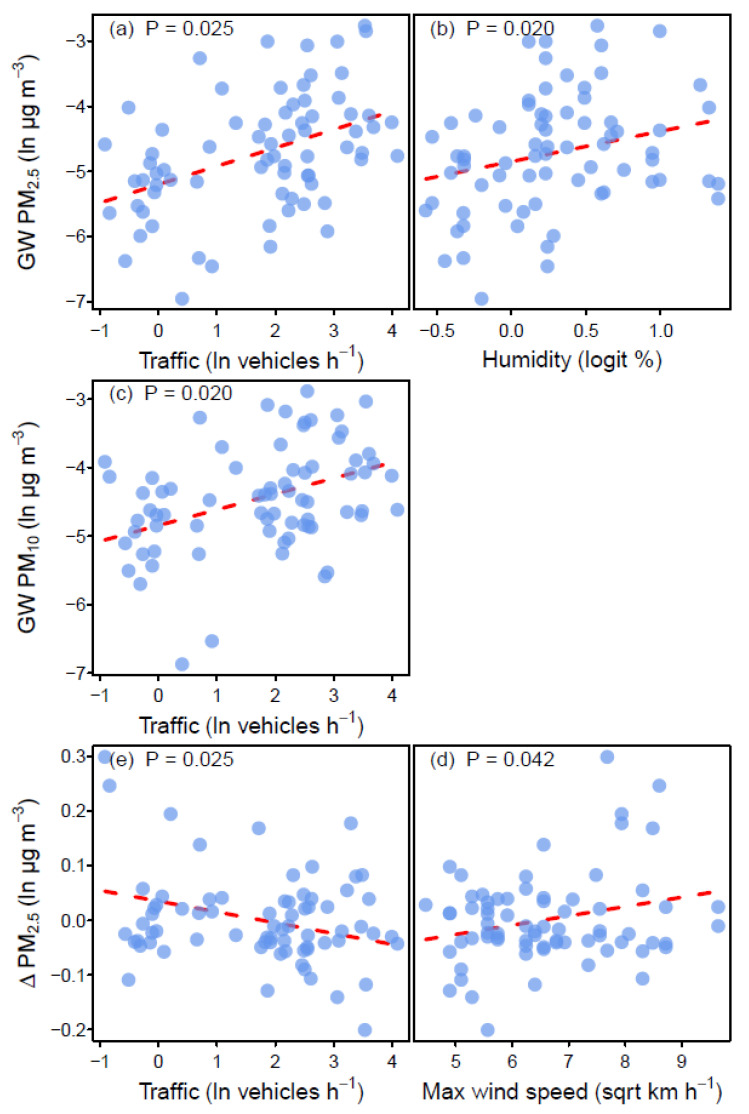
Scatterplots of the significant patterns emerging from the multiple regression models of (**a**) green wall PM_2.5_ and traffic, (**b**) green wall PM_2.5_ and humidity, (**c**) green wall PM_10_ and traffic, (**d**) ∆ PM_2.5_ and traffic, and (**e**), ∆ PM_10_ and max wind speed. Broken lines are coefficients for the model term of interest, with *p*-values for the terms shown at top left of each plot.

As ambient conditions, such as traffic and weather patterns, have been previously shown to have an influence on PM conditions, monthly traffic density and weather variables were recorded at each site. Analysis of these data indicated that higher green wall PM_2.5_ was significantly related to both greater traffic density and higher humidity (Table 2; Figure 4), while higher green wall PM_10_ was related only to traffic density (Table 2; Figure 4). This result was not surprising, as traffic density is well known to influence air pollution conditions [71,80]. A significant association between lower ∆ PM_2.5_ and heavier traffic, and higher maximum wind speed was found (Table 2; Figure 4), indicating that both these factors may affect the detectable pollutant removal effect of green walls, as has been found previously in pollutant distribution studies e.g., [65].

### 3.2. Differences in Noise and Temperature Conditions between Wall Types

**Figure 5:**
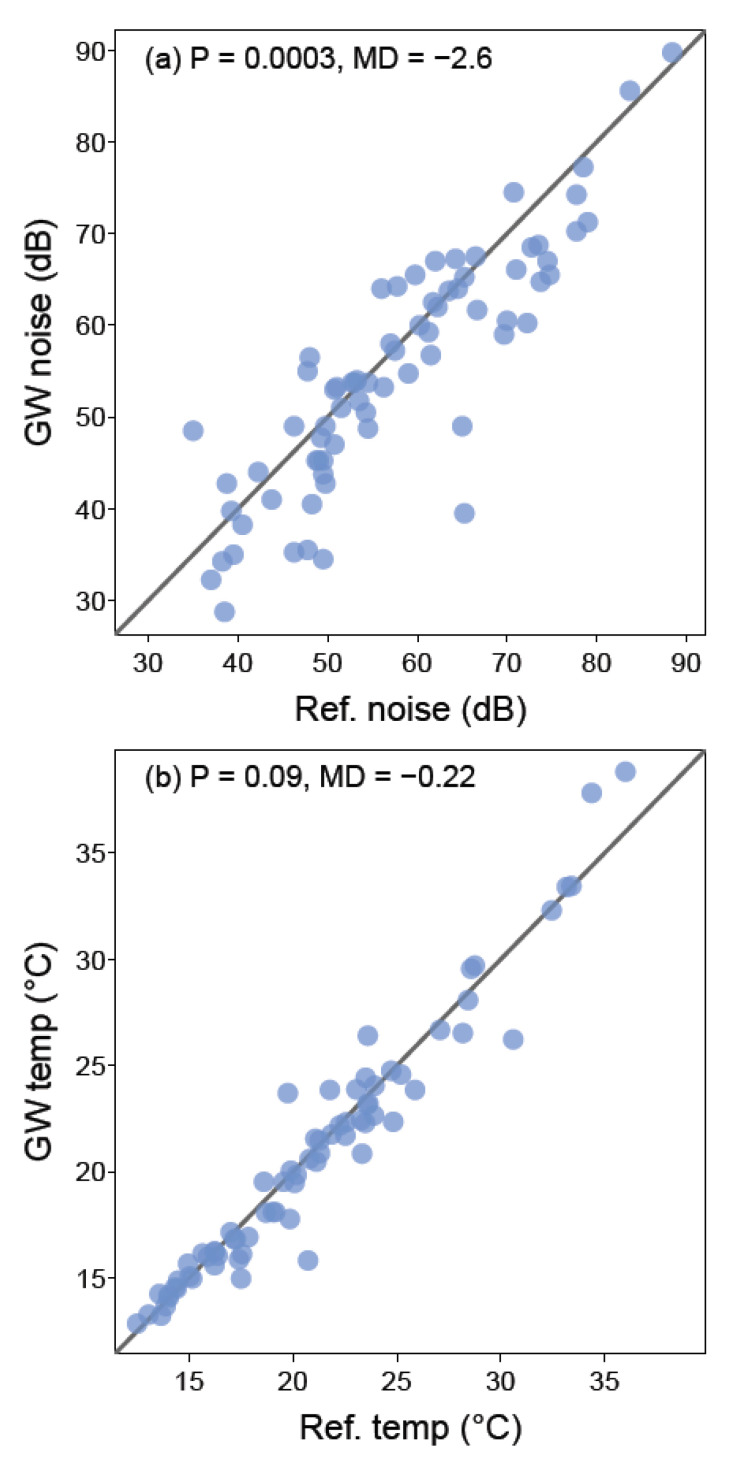
Scatter plots showing noise (**a**), and temperature (**b**) at the green wall (*y*-axis) and reference wall (*x*-axis). Black lines represent a 1:1 relationship, therefore all points falling below the line indicate lower values at the green wall relative to the reference wall, and the converse for points above the line. In both plots, *p*-values (paired *t*-test) and the mean difference (MD, green wall value—reference wall value) between paired samples in shown.

Ambient noise was significantly lower proximal to the green walls relative to their paired reference sites (t_71_ = −3.55, *p* = 0.0003; Figure 5a), while no significant difference in temperature was found between treatment groups (t_71_ = −1.34, *p* = 0.1; Figure 5b).

Whilst the capacity of green walls to reduce urban noise is still not well understood [7], it is known that plants can absorb noise more effectively than most hard surfaces, which reflect noise of all wavelengths [24,81]. Previous modelling studies have predicted the noise reduction capacity of green walls, estimating that they can reduce 2–5 dB (A) of single point source noises [82], and up to 1.6 dB (A) of road traffic noise [83]. The modelling study conducted by [84] predicted that green walls could reduce emergent and traffic noise by up to 10 dB (A). Klingberg et al. [71] found that traffic noise reduction was proportional to depth of vegetation through which the sound passed, with reductions of 0.6–2.3 dB recorded, a lower range than the site/month average of 1.34–6.40 dB detected in the current work. Interestingly, the maximum noise reduction observed in the current study was 12.13 dB, with this extreme value likely due to the specific and unusual noise type experienced in that sample (ocean related noise, i.e., waves crashing) compared to the predominantly traffic related noise at the other sites.

No significant temperature differences were observed in the current study between the green wall and reference walls, a surprising finding as plants are known to have an air cooling capacity resulting from evapotranspiration. However, whilst [85] recorded temperature reductions of 0.8–4.8 °C from an active green wall, and most indoor green wall studies have demonstrated significant effects on temperature [86,87], outdoor temperature reduction studies have produced variable findings. The work conducted by Alspach and Göhring [88], has indicted that green walls induced temperature reductions of up to 10 °C, with effects strongest in built environments with a height to width ratio greater than 2, which includes dense urban cities such as Melbourne and Hong Kong [52]. It is therefore possible that the building characteristics and unorthodox grid street design of Sydney precluded significant temperature reductions to be recorded in the current work. Alternatively, [89] noted that peak temperature reductions more significant than average reductions, with the effect being of most benefit during extreme heat waves. In the current study, peak temperature reductions were also observed; however, the reductions became non-significant when averaged across sites and months. As such, whilst the green walls within the current study were not effective at consistently reducing ambient temperatures, they still provided occasional peak reductions over the sampling period, and thus may provide benefits in variable climates such as those experiences in Sydney’s Summer periods.

**Figure 6:**
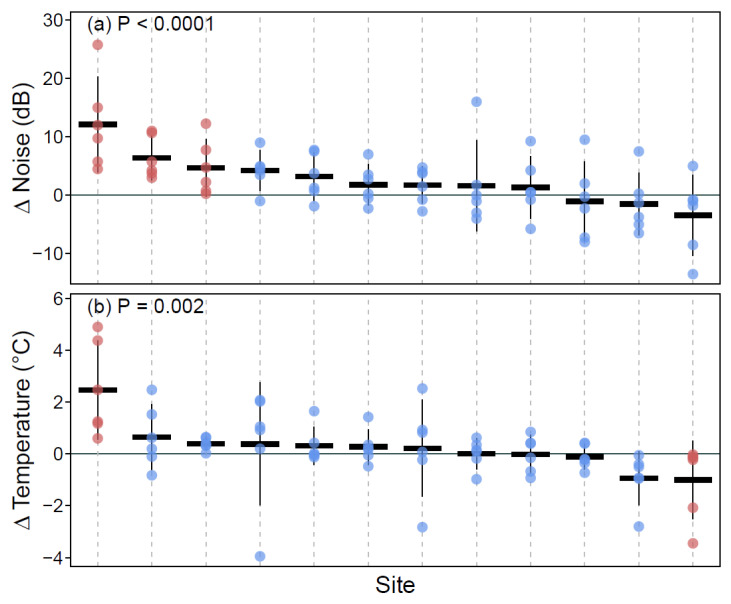
Plots of ∆ noise (**a**) and ∆ temperature (**b**). Red points are the individual sites that are significantly different from their respective reference sites, and blue points are sites where no significant site-wise differences were observed. Thick black horizontal lines indicate site means, and thin vertical black lines show the 95% confidence interval of the mean for the sites. The *p*-value at the top of each plot is the result from a joint test of model coefficients with the null hypothesis that the sites do not differ from zero. Sites are sorted on the *x*-axis by their mean value (highest to lowest) for ease in interpretation. The solid horizontal line indicates zero on the *y*-axis, representing equal values of noise (**a**) or temperature (**b**) at the green wall and reference wall.

Across sites, it was found that both ∆ noise (F_12,60_ = 4.82, *p* < 0.0001) and ∆ temperature (F_12,60_ = 3.02, *p* = 0.002) significantly differed from zero. At the site level, ∆ noise was found to be significantly greater than zero at four sites (i.e., noise at green wall sites was lower relative to the reference wall; Figure 6a), while ∆ temperature showed more equivocal results, with one site exhibiting ∆ temperature significantly more than zero, and one other site having ∆ temperature significantly less than zero (Figure 6b).

## 4. Conclusions

This study assessed the capacity of in situ passive green walls to reduce ambient PM pollution and noise, and to influence proximal temperature. No significant differences were observed between PM concentrations at the green wall and reference wall locations across the 12 sites; indicating that the current passive systems were not capable of reducing PM conditions to a level detectable by the methods used here. The green wall sites tested in the current study, however, had significantly lower proximal noise levels compared to the reference wall sites, indicating that the plants appeared to be absorbing ambient noise. Proximal ambient temperatures at the green wall and reference wall locations were not significantly different. It is thus possible that some previous studies that have used computational modelling procedures to predict major temperature and PM reductions from green walls may have overestimated the in situ effects.

## Figures and Tables

**Table 1:** Test site location descriptions

Site Number	Site Location	Notes	General Land Use	Elevation above Sea Level (m)	Size (m^2^)	Number of Plants
1	Ashfield (33°53′25.7″ S 151°07′40.3″ E)	Apartment complex with green wall in outdoor foyer	Residential	18	27	1296
2	Tamarama (33°53′53.5″ S 151°16′23.6″ E)	Residential property, green wall situated in back yard	Residential	33	12.5	600
3	Mosman (33°49′41.7″ S 151°14′04.1″ E)	Apartment complex with outdoor green wall	Residential and industry	70	72	3456
4	Lane Cove (33°48′55.8″ S 151°10′10.6″ E)	Display home with green wall situated in an outdoor area	Industry	50	9	432
5	Woollahra (33°53′15.7″ S 151°14′59.1″ E)	Residential property, green wall in front courtyard	Residential	85	6	288
6	Gordon (33°45′33.2″ S 151°09′20.0″ E)	High School. Green wall situated in a courtyard	Residential	121	140	9150
7	The Rocks, Site 1 (33°51′45.6″ S 151°12′20.4″ E)	Extensive green wall situated on expressway	Transport	19	142	6891
8	The Rocks, Site 2 (33°51′39.4″ S 151°12′29.9″ E)	Green wall situated under rail line support structure	Transport	19	25	1600
9	Summer Hill (33°53′29.7″ S 151°08′10.2″ E)	High School, green wall situated in a courtyard	Residential	55	4.5	216
10	Camperdown (33°53′04.0″ S 151°10′33.8″ E)	Multi-storey apartment complex	Residential and commercial	30	18	864
11	Ultimo (33°53′00.7″ S 151°11′58.0″ E)	Green wall situated on a tertiary education facility	Educational	15	145	9280
12	Crows Nest (33°49′38.3″ S 151°12′07.0″ E)	Green wall situated on the exterior of a grocery store	Commercial	101	25	1200

**Table 2:** Results from multiple regression LMMs of green wall PM_2.5_ and PM_10_, ∆ PM_2.5_, and ∆ PM_10_. Bold ***p*** values indicate significant relationship.

Response	Terms	Estimate	SE	df	t Value	*p*
Green wall PM_2.5_	Traffic	0.281	0.101	7.8	2.782	**0.03**
	Max wind speed	−0.139	0.077	62.2	−1.811	0.07
	Humidity	0.465	0.194	62.3	2.391	**0.02**
	Green wall area	−1.198	1.214	6.4	−0.986	0.40
	Plant number	1.173	1.125	6.4	1.042	0.30
Green wall PM_10_	Traffic	0.23	0.081	8.6	2.847	**0.02**
	Max wind speed	−0.098	0.073	64.5	−1.334	0.20
	Humidity	0.197	0.185	64.6	1.062	0.30
	Green wall area	−0.389	0.955	7.4	−0.407	0.70
	Plant number	0.333	0.885	7.4	0.376	0.70
∆ PM_2.5_	Traffic	−0.02	0.009	66	−2.299	**0.03**
	Max wind speed	0.017	0.008	66	2.076	**0.04**
	Humidity	0.002	0.021	66	0.081	0.90
	Green wall area	0.079	0.101	66	0.778	0.40
	Plant number	−0.071	0.094	66	−0.758	0.50
∆ PM_10_	Traffic	0.004	0.007	66	0.628	0.50
	Max wind speed	−0.004	0.007	66	−0.497	0.60
	Humidity	0.02	0.018	66	1.131	0.30
	Green wall area	−0.053	0.088	66	−0.604	0.50
	Plant number	0.05	0.081	66	0.62	0.50

## Appendix A

**Table A1:** Test sites with a visual aid.

Site Number	Site Location	Picture
1	Ashfield	
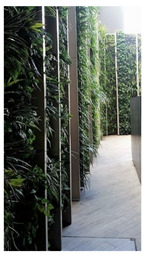
2	Tamarama	
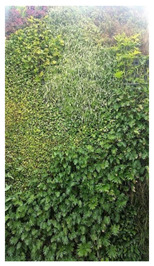
3	Mosman	
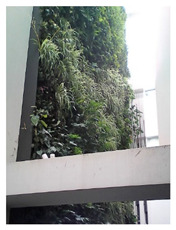
4	Lane Cove	
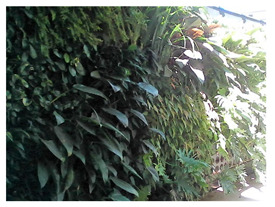
5	Woollahra	
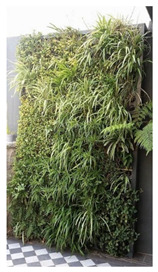
6	Gordon	
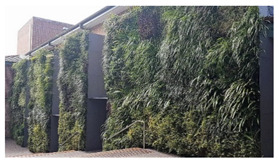
7	The Rocks, Site 1	
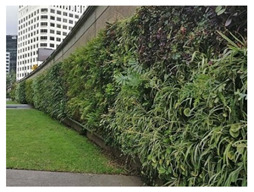
8	The Rocks, Site 2	
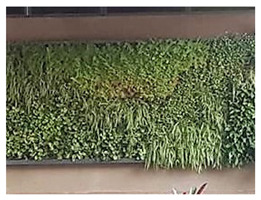
9	Summer Hill	
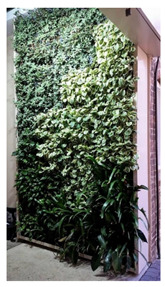
10	Camperdown	
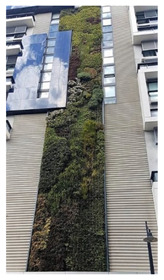
11	Ultimo	
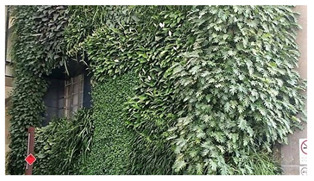
12	Crows Nest	
